# Consumption of spicy foods and total and cause specific mortality: population based cohort study

**DOI:** 10.1136/bmj.h3942

**Published:** 2015-08-04

**Authors:** Jun Lv, Lu Qi, Canqing Yu, Ling Yang, Yu Guo, Yiping Chen, Zheng Bian, Dianjianyi Sun, Jianwei Du, Pengfei Ge, Zhenzhu Tang, Wei Hou, Yanjie Li, Junshi Chen, Zhengming Chen, Liming Li

**Affiliations:** 1 Department of Epidemiology and Biostatistics, School of Public Health, Peking University Health Science Center, Beijing 100191, People’s Republic of China; 2 Department of Nutrition, Harvard School of Public Health, Boston, MA, USA; 3 Channing Division of Network Medicine, Department of Medicine, Brigham and Women’s Hospital and Harvard Medical School, Boston, MA, USA; 4 Clinical Trial Service Unit and Epidemiological Studies Unit, Nuffield Department of Population Health, University of Oxford, UK; 5 Chinese Academy of Medical Sciences, Beijing, People’s Republic of China; 6 Hainan Center for Disease Control and Prevention, Haikou, Hainan, People’s Republic of China; 7 Gansu Center for Disease Control and Prevention, Lanzhou, Gansu, People’s Republic of China; 8 Guangxi Center for Disease Control and Prevention, Liuzhou, Guangxi, People’s Republic of China; 9 Licang Center for Disease Control and Prevention, Qingdao, Shandong, People’s Republic of China; 10 Nangang Center for Disease Control and Prevention, Harbin, Heilongjiang, People’s Republic of China; 11 China National Center for Food Safety Risk Assessment, Beijing, People’s Republic of China

## Abstract

**Objective** To examine the associations between the regular consumption of spicy foods and total and cause specific mortality.

**Design** Population based prospective cohort study.

**Setting** China Kadoorie Biobank in which participants from 10 geographically diverse areas across China were enrolled between 2004 and 2008.

**Participants **199 293 men and 288 082 women aged 30 to 79 years at baseline after excluding participants with cancer, heart disease, and stroke at baseline.

**Main exposure measures** Consumption frequency of spicy foods, self reported once at baseline.

**Main outcome measures** Total and cause specific mortality.

**Results** During 3 500 004 person years of follow-up between 2004 and 2013 (median 7.2 years), a total of 11 820 men and 8404 women died. Absolute mortality rates according to spicy food consumption categories were 6.1, 4.4, 4.3, and 5.8 deaths per 1000 person years for participants who ate spicy foods less than once a week, 1 or 2, 3 to 5, and 6 or 7 days a week, respectively. Spicy food consumption showed highly consistent inverse associations with total mortality among both men and women after adjustment for other known or potential risk factors. In the whole cohort, compared with those who ate spicy foods less than once a week, the adjusted hazard ratios for death were 0.90 (95% confidence interval 0.84 to 0.96), 0.86 (0.80 to 0.92), and 0.86 (0.82 to 0.90) for those who ate spicy food 1 or 2, 3 to 5, and 6 or 7 days a week, respectively. Compared with those who ate spicy foods less than once a week, those who consumed spicy foods 6 or 7 days a week showed a 14% relative risk reduction in total mortality. The inverse association between spicy food consumption and total mortality was stronger in those who did not consume alcohol than those who did (P=0.033 for interaction). Inverse associations were also observed for deaths due to cancer, ischemic heart diseases, and respiratory diseases.

**Conclusion** In this large prospective study, the habitual consumption of spicy foods was inversely associated with total and certain cause specific mortality, independent of other risk factors of death.

## Introduction

Spices have been an integral part of culinary cultures around the world and have a long history of use for flavoring, coloring, and preserving food, as well as for medicinal purposes. The increased use of spices as flavorings in foods is a major trend worldwide.^1^ ^2^ In China, chilli pepper is among the most popular spicy foods consumed nationwide.^3^

The beneficial effects of spices and their bioactive ingredients such as capsaicin have long been documented in experimental or small sized population studies. For example, an ecological study showed that populations with a higher consumption of spices have a lower incidence of cancer.^4^ The ingestion of red pepper was found to decrease appetite and energy intake in people of Asian origin and white people and might reduce the risk of overweight and obesity.^5^ ^6^ In addition, the bioactive agents in spices have also shown beneficial roles in obesity, cardiovascular and gastrointestinal conditions, various cancers, neurogenic bladder, and dermatological conditions.^7^ ^8^ ^9^ Moreover, spices exhibit antibacterial activity and affect gut microbiota populations, which in humans have been recently related to risks of diabetes, cardiovascular disease, liver cirrhosis, and cancer.^10^ ^11^ ^12^ These data collectively suggest that spices may have a profound influence on morbidities and mortality in humans; however, the evidence relating daily consumption of spicy foods and total and disease specific mortality from population studies is lacking.

We prospectively examined the associations of the regular consumption of spicy foods in a daily diet with total and cause specific mortality in the China Kadoorie Biobank (CKB) study of 0.5 million adults.^13^ ^14^

## Methods

### Study population

The China Kadoorie Biobank is a prospective cohort study of over 0.5 million adults from 10 geographically diverse areas across China; participants were enrolled between 2004 and 2008 and have been followed up ever since for morbidities and mortality. Further details of the China Kadoorie Biobank study have been described elsewhere.^13^ ^14^ Briefly, a total of 512 891 adults aged 30-79 years had valid baseline data—that is, completed questionnaire, physical measurements, and a written informed consent form.

In the present study we excluded 2577 people with cancer, 15 472 with heart disease, and 8884 with stroke at baseline, as well as three people with a recorded implausible censoring date for loss to follow-up. The final analyses included 199 293 men and 288 082 women. All participants provided information on spicy food consumption. All gave informed consent before taking part.

### Patient involvement

There was no patient involvement in this study.

### Assessment of spicy food consumption

In the baseline questionnaire we asked the participants “During the past month, about how often did you eat hot spicy foods?”: never or almost never, only occasionally, 1 or 2 days a week, 3 to 5 days a week, or 6 or 7 days a week. The participants who selected the last three categories were further asked “When you eat spicy foods, what are the main sources of spices usually used?” (multiple choices allowed fresh chilli pepper, dried chilli pepper, chilli sauce, chilli oil, and other or don’t know).

After completion of the baseline survey in July 2008, about 5% randomly chosen surviving participants in 10 survey sites were resurveyed during August and October of 2008.^14^ To test the reproducibility of the frequency of spicy food consumption, we included 1300 participants who completed the same questionnaire twice at an interval of less than 1.5 years (median 1.4 years). Spearman’s coefficient for the correlation between the two questionnaires was 0.71, indicating that spicy food consumption was reported consistently.

### Assessment of covariates

We obtained covariates from the baseline questionnaire, including sociodemographic characteristics (age, sex, education, occupation, household income, and marital status), lifestyle behaviors (alcohol consumption, tobacco smoking, physical activity, and intakes of red meat, fresh fruits, and vegetables), personal health and medical history (hypertension, diabetes, chronic hepatitis or cirrhosis, peptic ulcer, gallstones or cholecystitis, and menopausal status for women only), and information on family members, including biological parents and siblings who had had cancer, heart attack, stroke, or diabetes. The daily level of physical activity was calculated by multiplying the metabolic equivalent tasks (METs) value for a particular type of physical activity by hours spent on that activity per day and summing the MET hours for all activities. Habitual dietary intake in the past year was assessed by a qualitative food frequency questionnaire. A participant was considered as having a family history of a particular disease if they reported at least one first degree relative with the disease.

At baseline, trained staff measured body weight, height, and blood pressure using calibrated instruments. Body mass index was calculated as weight (kg)/(height (m)^2^. A stepwise on-site testing of plasma glucose level was undertaken using the SureStep Plus meter (LifeScan; Milpitas, CA). Prevalent hypertension was defined as a measured systolic blood pressure of 140 mm Hg or more, a measured diastolic blood pressure of 90 mm Hg or more, self reported diagnosis of hypertension, or self reported use of antihypertensive drugs at baseline. Prevalent diabetes was defined as a measured fasting blood glucose concentration of 7.0 mmol/L or more, a measured random blood glucose concentration of 11.1 mmol/L or more, or self reported diagnosis of diabetes.

### Ascertainment of deaths

We ascertained vital status by means of linkage with local disease surveillance points system death registries^15^ and residential records. To minimize the under-reporting of deaths and to identify participants who had moved permanently out of the study areas, we also carried out separate active follow-up annually by reviewing residential records, visiting local communities, or directly contacting participants.^14^ The causes of death were sought chiefly from official death certificates that were supplemented, if necessary, by a review of the medical records or undertaking a verbal autopsy using a validated instrument for those with an ill defined or unknown cause of death reported. The electronic linkage to the national health insurance claim databases started in 2011, which has become an important means of follow-up and helped to improve the accuracy of diagnosis and phenotyping of reported conditions, outcome adjudication, and further data collection. Linkage to local health insurance databases has been achieved for about 95% of the participants in 2013. Participants from both urban and rural areas had similar proportions of successful linkage to health insurance databases. Linkage to local health insurance database was renewed annually. Participants who failed to be linked to local health insurance database were actively followed annually by staff to ascertain their status, including hospital admission, death, and moving out of the study area. Linkage to a local health insurance database has become an important supplementary way of ascertaining deaths.

Trained staff blinded to baseline information classified any deaths occurring among participants by using ICD-10 codes (international classification of diseases, 10th revision). The deaths were grouped into seven categories: cancer (C00-C97), ischemic heart diseases (I20-I25), cerebrovascular diseases (I60-I69), diabetes mellitus (E10-E14), diseases of the respiratory system (J00-J99), infections (A00-B99), and all other causes. Losses to follow-up in this study refer to participants whose permanent registered residence was no longer in the study area, who could not be contacted after at least three reasonable efforts within one year, or who could be contacted but their new residence was out of the jurisdiction of the regional coordinating center.

### Statistical analysis

We measured person years from baseline (2004–08) until the date of death, loss to follow-up, or 31 December 2013, whichever occurred first. Cox proportional hazards regression models were used to estimate the hazard ratios and 95% confidence intervals of mortality for spicy food consumption, with age as the underlying time scale. We accounted for the group specific effect of 10 survey sites on the hazard function by stratifying on the survey site variable in the Cox model.

Multivariate models were adjusted for established and potential risk factors for death: age (continuous, serving as the underlying timescale); sex (male or female); level of education (no formal school, primary school, middle school, high school, college, or university or higher); marital status (married, widowed, divorced or separated, or never married); alcohol consumption (non-drinker, occasional drinker, former drinker, or regular drinker); smoking status (never smoker, occasional smoker, former smoker, or regular smoker); physical activity in MET hours a day (continuous); body mass index (continuous); intake frequencies of red meat, fresh fruits, and vegetables (daily, 4-6 days/wk, 1-3 days/wk, monthly, or rarely or never); prevalent hypertension and diabetes at baseline (presence or absence); menopausal status (for women only, premenopausal, perimenopausal, or postmenopausal); and status of family history of cancer, heart attack, stroke, or diabetes (presence or absence). We adjusted for the family history variables only in the corresponding analysis of cause specific mortality.

To examine the robustness of our findings, we also conducted several sensitivity analyses: additionally adjusting for occupation and household income; additionally adjusting for histories of chronic hepatitis or cirrhosis, peptic ulcer, and gallstone or cholecystitis; adjusting for a 13 level detailed smoking variable, which incorporated the information on amount of smoking in regular smokers and the time since quitting in former smokers, instead of a four level smoking variable; excluding participants dying during the first two years of follow-up; excluding participants who had diabetes at baseline; excluding participants who reported exclusive use of other spices instead of any types of chilli; stratifying analyses by rural or urban residence; and stratifying analyses by follow-up duration (<3 or ≥3 years).

Subgroup analyses were conducted separately among participants who did and did not report fresh chilli pepper as one of their commonly used spices. We compared both with those who ate spicy foods less than once a week. We also examined the associations of spicy food consumption with total mortality among prespecified baseline subgroups based on age (<50, 50 to 59, or ≥60), smoking status (regular smoker, or not), alcohol consumption (regular drinker, or not), level of physical activity (categorized using tertile cut-offs), and body mass index (<24.0, 24.0 to 27.9, or ≥28.0). The tests for interaction were performed by means of likelihood ratio tests, which involved comparing models with and without cross product terms between the baseline stratifying variable and spicy food consumption as an ordinal variable.

The statistical analyses were performed with Stata (version 13.1, Stata). All P values were two sided, and we defined statistical significance as P<0.05.

## Results

### Spicy food consumption and lifestyle and dietary factors

Table 1 presents the age and site adjusted baseline characteristics of the participants according to the categories of spicy food consumption. Compared with participants who consumed spicy foods less frequently (3 to 5 days a week or less), those who consumed spicy foods almost every day were more likely to be rural residents, more likely to smoke tobacco and consume alcohol, and more frequently to consume red meat, vegetables, and fruits. Fresh and dried chilli peppers were the most commonly used types of spices in those who reported consuming spicy foods weekly (table 1).

**Table 1 tbl1:** Baseline characteristics of the study participants according to weekly spicy food consumption. Values are numbers (percentages) of participants unless stated otherwise

Characteristics	Men (n=199 293)					Women (n=288 082)			
	Less than once a week	1 or 2 days	3-5 days	6 or 7 days		Less than once a week	1 or 2 days	3-5 days	6 or 7 days
No of participants	110 995	14 217	12 732	61 349		167 496	17 523	15 817	87 246
Mean age (years)	52.9	49.3	49.2	51.1		51.9	48.1	48.1	48.8
Rural area	53 076 (47.8)	5847 (41.2)	5089 (40.0)	50 360 (82.1)		76 404 (46.0)	7588 (42.7)	6889 (43.0)	71 982 (82.3)
Married	103 512 (92.6)	13 387 (93.0)	11 998 (93.2)	56 406 (93.4)		147 937 (89.3)	15 780 (88.7)	14 258 (88.7)	79 663 (90.0)
Middle school and higher	65 920 (56.5)	9491 (59.6)	8640 (59.9)	31 102 (59.0)		71 789 (41.5)	9238 (45.5)	8573 (46.8)	34 921 (45.3)
Mean body mass index	23.2	23.5	23.6	23.6		23.6	23.7	23.9	24.1
Diabetes	6483 (5.1)	761 (5.1)	669 (5.0)	2226 (5.1)		10 842 (5.5)	862 (5.5)	765 (5.5)	3554 (5.7)
Hypertension	43 038 (34.7)	5074 (37.1)	4431 (37.2)	19 031 (37.9)		59 239 (31.8)	4777 (31.7)	4216 (31.7)	24 532 (33.2)
Postmenopausal	—	—	—	—		93 125 (51.2)	7254 (50.7)	6485 (50.2)	39 292 (50.0)
Family medical history:									
Cancer	24 557 (19.7)	2941 (20.2)	2675 (20.9)	9827 (20.7)		35 063 (18.5)	3372 (19.1)	3184 (20.3)	12 440 (19.0)
Stroke	24 243 (20.3)	3014 (20.5)	3003 (22.7)	11 402 (21.9)		34 886 (19.2)	3682 (20.3)	3621 (22.1)	15 022 (20.9)
Heart attack	7259 (6.3)	911 (6.4)	833 (6.5)	4093 (7.1)		9798 (5.5)	1047 (5.8)	1050 (6.4)	4608 (6.2)
Diabetes	11 620 (9.7)	1571 (10.2)	1416 (10.3)	5252 (10.5)		17 427 (9.2)	2033 (10.7)	1882 (11.1)	6400 (10.1)
Regular smoker	62 767 (57.0)	9255 (63.1)	8534 (65.6)	43 225 (70.4)		2715 (1.8)	383 (2.1)	444 (2.7)	3048 (3.0)
Regular drinker	33 788 (27.0)	5778 (36.6)	5588 (40.6)	22 486 (47.2)		2171 (1.2)	444 (2.3)	500 (2.9)	2888 (3.8)
Mean physical activity (MET h/day)	22.6	22.3	22.6	22.7		20.6	20.9	21.2	21.1
Average weekly consumption*:									
Red meat (day)	3.8	3.9	3.9	4.2		3.4	3.4	3.5	3.7
Fresh vegetables (day)	6.8	6.5	6.7	7.0		6.8	6.4	6.7	7.0
Fresh fruits (day)	2.0	2.0	1.9	2.2		2.4	2.6	2.5	2.9
Commonly used types of spices†:									
Fresh chilli pepper	—	8904 (76.3)	8743 (79.4)	54 546 (84.4)		—	10 745 (73.4)	10 768 (77.3)	76 819 (84.9)
Dried chilli pepper	—	5852 (55.8)	6223 (62.5)	48 486 (75.4)			6067 (50.0)	7082 (58.3)	67 372 (74.2)
Chilli sauce	—	6106 (39.2)	5921 (40.9)	25 406 (44.7)		—	7609 (40.2)	7278 (41.6)	37 621 (45.6)
Chilli oil	—	6959 (40.5)	6923 (42.6)	25 160 (46.2)		—	8553 (41.1)	8517 (43.5)	38 226 (47.8)
Other or don’t know	—	3735 (18.7)	3375 (19.6)	13 886 (27.0)		—	4176 (16.9)	3746 (17.5)	20 091 (26.7)

MET=metabolic equivalent of task.

All variables were adjusted for age and survey sites, as appropriate. All exposures were associated with spicy food consumption, with P<0.001 for trends across categories, except for diabetes (men: P=0.872; women: P=0.186), family history of cancer in women (P=0.002), and physical activity in men (P=0.546). Tests for linear trend across categories were performed by assigning the midpoint values of each spicy food consumption category and treating the variable as continuous in a separate regression model.

*Average weekly consumptions of red meat, fresh vegetables, and fruits were calculated by assigning participants to the midpoint of their consumption category.

†Among those eating spicy foods at least once a week.

### Spicy food consumption and total mortality

During a median follow-up of 7.2 years (interquartile range 1.84 years; total person years 3 500 004), we documented 11 820 deaths among men and 8404 deaths among women. Absolute mortality rates according to spicy food consumption categories were 6.1, 4.4, 4.3, and 5.8 deaths per 1000 person years for participants who ate spicy foods less than once a week and 1 or 2, 3 to 5, and 6 or 7 days a week, respectively. Age adjusted and multivariate adjusted analyses showed a statistically significant inverse association between spicy food consumption and total mortality. In the whole cohort, compared with participants who ate spicy foods less than once a week, the adjusted hazard ratios for death were 0.90 (95% confidence interval 0.84 to 0.96) for those who ate spicy foods 1 or 2 days a week, 0.86 (0.80 to 0.92) for 3 to 5 days a week, and 0.86 (0.82 to 0.90) for 6 or 7 days a week (table 2). Compared with participants who ate spicy foods less than once a week, those who consumed spicy foods 6 or 7 days a week showed a 14% relative risk reduction in total mortality. The multivariate adjusted hazard ratios for total mortality among men, compared with men who ate spicy foods less than once a week, were 0.90 (0.83 to 0.98) for those who ate spicy food 1 or 2 days a week, 0.90 (0.83 to 0.99) for 3 to 5 days, and 0.90 (0.85 to 0.96) for 6 or 7 days a week; the respective hazard ratios among women were 0.88 (0.79 to 0.98), 0.78 (0.69 to 0.88), and 0.81 (0.75 to 0.87) (table 3). There was no heterogeneity between men and women in any of the associations (P=0.723).

**Table 2 tbl2:** Association of weekly spicy food consumption with total and cause specific mortality among 487 375 participants. Values are hazard ratios (95% CIs) unless stated otherwise

Cause of death	No of participants	Frequency of spicy food consumption			
		Less than once a week*	1 or 2 days	3-5 days	6 or 7 days
No of participants	487 375	278 491	31 740	28 549	148 595
No of person years	3 500 004	1 990 589	228 112	204 972	1 076 330
All causes:					
No of deaths†	20 224	12 145	1014	876	6189
Model 1		1.00	0.90 (0.84 to 0.96)	0.85 (0.79 to 0.91)	0.83 (0.79 to 0.86)
Model 2		1.00	0.89 (0.83 to 0.95)	0.84 (0.79 to 0.90)	0.82 (0.79 to 0.86)
Model 3		1.00	0.90 (0.84 to 0.96)	0.86 (0.80 to 0.92)	0.86 (0.82 to 0.90)
Cancer:					
No of deaths†	7256	4636	378	349	1893
Model 1		1.00	0.96 (0.86 to 1.07)	1.01 (0.90 to 1.13)	0.95 (0.88 to 1.03)
Model 2		1.00	0.91 (0.81 to 1.01)	0.95 (0.85 to 1.06)	0.89 (0.83 to 0.96)
Model 3		1.00	0.92 (0.83 to 1.03)	0.97 (0.87 to 1.08)	0.92 (0.85 to 0.99)
Ischemic heart diseases:					
No of deaths†	2302	1406	109	93	694
Model 1		1.00	0.83 (0.68 to 1.01)	0.75 (0.60 to 0.93)	0.73 (0.64 to 0.84)
Model 2		1.00	0.84 (0.69 to 1.03)	0.76 (0.61 to 0.94)	0.75 (0.65 to 0.86)
Model 3		1.00	0.82 (0.67 to 1.00)	0.75 (0.61 to 0.94)	0.78 (0.67 to 0.89)
Cerebrovascular diseases:					
No of deaths†	4024	2217	215	170	1422
Model 1		1.00	1.01 (0.88 to 1.17)	0.84 (0.72 to 0.99)	0.92 (0.83 to 1.01)
Model 2		1.00	1.03 (0.89 to 1.19)	0.86 (0.73 to 1.01)	0.94 (0.85 to 1.04)
Model 3		1.00	1.03 (0.89 to 1.20)	0.86 (0.73 to 1.01)	0.96 (0.87 to 1.07)
Diabetes:					
No of deaths†	569	328	30	17	194
Model 1		1.00	0.77 (0.52 to 1.13)	0.46 (0.28 to 0.75)	0.59 (0.46 to 0.76)
Model 2		1.00	0.87 (0.59 to 1.27)	0.52 (0.32 to 0.86)	0.70 (0.55 to 0.90)
Model 3		1.00	0.94 (0.64 to 1.40)	0.60 (0.36 to 0.99)	0.82 (0.63 to 1.05)
Respiratory diseases:					
No of deaths†	1996	1203	77	70	646
Model 1		1.00	0.60 (0.48 to 0.76)	0.59 (0.46 to 0.76)	0.57 (0.50 to 0.66)
Model 2		1.00	0.64 (0.51 to 0.81)	0.62 (0.49 to 0.80)	0.63 (0.55 to 0.72)
Model 3		1.00	0.67 (0.53 to 0.85)	0.65 (0.51 to 0.83)	0.71 (0.62 to 0.81)
Infections:					
No of deaths†	336	187	20	15	114
Model 1		1.00	0.87 (0.54 to 1.39)	0.68 (0.40 to 1.17)	0.71 (0.51 to 0.98)
Model 2		1.00	0.90 (0.56 to 1.45)	0.72 (0.42 to 1.23)	0.77 (0.55 to 1.06)
Model 3		1.00	0.91 (0.56 to 1.48)	0.74 (0.43 to 1.28)	0.83 (0.60 to 1.15)
All other causes:					
No of deaths†	3741	2168	185	162	1226
Model 1		1.00	0.90 (0.77 to 1.05)	0.89 (0.75 to 1.04)	0.81 (0.73 to 0.90)
Model 2		1.00	0.89 (0.76 to 1.04)	0.88 (0.75 to 1.04)	0.81 (0.73 to 0.91)
Model 3		1.00	0.89 (0.77 to 1.04)	0.89 (0.75 to 1.05)	0.86 (0.77 to 0.95)

Multivariate models were adjusted for: model 1: age (years); model 2: additionally included sex (male or female); level of education (no formal school, primary school, middle school, high school, college, or university or higher); marital status (married, widowed, divorced or separated, or never married); alcohol consumption (non-drinker, occasional drinker, former drinker, or regular drinker); smoking status (never smoker, occasional smoker, former smoker, or regular smoker); physical activity (MET (metabolic equivalent of task) h/day); model 3: additionally included body mass index; intake frequencies of red meat, fruits, and vegetables (daily, 4 to 6 days/wk, 1 to 3 days/wk, monthly, or rarely or never); prevalent hypertension and diabetes at baseline (presence or absence); and family history of cancer, heart attack, stroke, or diabetes (presence or absence, only adjusted for in corresponding analysis of cause specific mortality).

*Reference group.

†During follow-up.

**Table 3 tbl3:** Association of weekly spicy food consumption with total and cause specific mortality by sex. Values are hazard ratios (95% CIs) unless stated otherwise

Cause of death	Frequency of spicy food consumption			
	Less than once a week*	1 or 2 days	3-5 days	6 or 7 days
**Men**				
No of participants	110 995	14 217	12 732	61 349
No of person years	783 656	101 892	90 478	440 002
All causes:				
No of deaths†	6872	606	545	3797
Multivariate adjusted hazard ratio (95% CI)	1.00	0.90 (0.83 to 0.98)	0.90 (0.83 to 0.99)	0.90 (0.85 to 0.96)
Cancer:				
No of deaths†	2769	230	236	1188
Multivariate adjusted hazard ratio (95% CI)	1.00	0.88 (0.77 to 1.01)	1.05 (0.92 to 1.21)	0.94 (0.85 to 1.04)
Ischemic heart diseases:				
No of deaths†	745	66	60	417
Multivariate adjusted hazard ratio (95% CI)	1.00	0.87 (0.67 to 1.13)	0.85 (0.65 to 1.11)	0.85 (0.70 to 1.02)
Cerebrovascular diseases:				
No of deaths†	1199	117	85	821
Multivariate adjusted hazard ratio (95% CI)	1.00	1.00 (0.82 to 1.22)	0.77 (0.62 to 0.97)	0.95 (0.83 to 1.09)
Diabetes:				
No of deaths†	116	13	7	95
Multivariate adjusted hazard ratio (95% CI)	1.00	1.10 (0.60 to 2.01)	0.71 (0.33 to 1.55)	0.99 (0.67 to 1.46)
Respiratory diseases:				
No of deaths†	706	51	38	422
Multivariate adjusted hazard ratio (95% CI)	1.00	0.79 (0.59 to 1.06)	0.61 (0.44 to 0.86)	0.81 (0.67 to 0.97)
Infections:				
No of deaths†	116	13	11	82
Multivariate adjusted hazard ratio (95% CI)	1.00	0.91 (0.50 to 1.65)	0.83 (0.44 to 1.58)	0.99 (0.67 to 1.48)
All other causes:				
No of deaths†	1221	116	108	772
Multivariate adjusted hazard ratio (95% CI)	1.00	0.92 (0.75 to 1.11)	0.97 (0.79 to 1.19)	0.89 (0.78 to 1.03)
**Women**				
No of participants	167 496	17 523	15 817	87 246
No of person years	1 206 933	126 220	114 494	636 329
All causes:				
No of deaths†	5273	408	331	2392
Multivariate adjusted hazard ratio (95% CI)	1.00	0.88 (0.79 to 0.98)	0.78 (0.69 to 0.88)	0.81 (0.75 to 0.87)
Cancer:				
No of deaths†	1867	148	113	705
Multivariate adjusted hazard ratio (95% CI)	1.00	0.97 (0.82 to 1.15)	0.82 (0.68 to 1.00)	0.87 (0.77 to 0.99)
Ischemic heart diseases:				
No of deaths†	661	43	33	277
Multivariate adjusted hazard ratio (95% CI)	1.00	0.74 (0.54 to 1.02)	0.63 (0.44 to 0.90)	0.70 (0.56 to 0.86)
Cerebrovascular diseases:				
No of deaths†	1018	98	85	601
Multivariate adjusted hazard ratio (95% CI)	1.00	1.08 (0.87 to 1.34)	0.96 (0.76 to 1.21)	0.98 (0.85 to 1.14)
Diabetes:				
No of deaths†	212	17	10	99
Multivariate adjusted hazard ratio (95% CI)	1.00	0.87 (0.52 to 1.46)	0.54 (0.28 to 1.04)	0.72 (0.52 to 1.01)
Respiratory diseases:				
No of deaths†	497	26	32	224
Multivariate adjusted hazard ratio (95% CI)	1.00	0.51 (0.34 to 0.76)	0.69 (0.48 to 1.00)	0.62 (0.51 to 0.76)
Infections:				
No of deaths†	71	7	4	32
Multivariate adjusted hazard ratio (95% CI)	1.00	0.86 (0.38 to 1.96)	0.55 (0.19 to 1.56)	0.55 (0.31 to 0.99)
All other causes:				
No of deaths†	947	69	54	454
Multivariate adjusted hazard ratio (95% CI)	1.00	0.85 (0.66 to 1.10)	0.76 (0.58 to 1.02)	0.82 (0.69 to 0.96)

Multivariate models were adjusted for several baseline factors: age (years); level of education (no formal school, primary school, middle school, high school, college, or university or higher); marital status (married, widowed, divorced or separated, or never married); alcohol consumption (non-drinker, occasional drinker, former drinker, or regular drinker); smoking status (never smoker, occasional smoker, former smoker, or regular smoker); physical activity (MET (metabolic equivalent of task) h/day); body mass index; intake frequencies of red meat, fruits, and vegetables (daily, 4 to 6 days/wk, 1 to 3 days/wk, monthly, or rarely/never); prevalent hypertension and diabetes at baseline (presence or absence); family history of cancer, heart attack, stroke, or diabetes (presence or absence, only adjusted for in corresponding analysis of cause specific mortality); and menopausal status (premenopausal, perimenopausal, or postmenopausal, for women only).

*Reference group.

†During follow-up.

### Spicy food consumption and cause specific mortality

After multivariate adjustment, spicy food consumption was inversely associated with the risks of death due to cancer, ischemic heart diseases, and respiratory diseases in the whole cohort (table 2). No statistically significant heterogeneity was observed in the associations between spicy food consumption and cause specific mortality by sex (all P>0.05). Nevertheless, the associations seemed to be less evident in men than in women (table 3). In addition, more frequent consumption of spicy foods in women was also significantly associated with a reduced risk of death due to infections.

### Sensitivity analyses

In the sensitivity analyses, the associations of spicy food consumption with total and cause specific mortality did not change appreciably with additional adjustment for occupation and household income; or additional adjustment for histories of chronic hepatitis or cirrhosis, peptic ulcer, and gallstone or cholecystitis; or adjustment for a 13 level detailed smoking variable instead of a four level smoking variable; or excluding participants dying during the first two years of follow-up; or excluding participants with prevalent diabetes at baseline; or excluding participants with exclusive use of other spices instead of any types of chilli (data not shown). The associations of spicy food consumption with total and cause specific mortality were consistently observed in participants from both rural and urban areas and for different follow-up durations (<3 or ≥3 years).

### Subgroup analyses

We further performed stratified analyses according to whether the participants reported using fresh chilli pepper as their predominant spice. We found that the inverse associations of daily spicy food consumption with death due to cancer, ischemic heart diseases, and diabetes seemed stronger in the fresh chilli group than in the non-fresh chilli group in the whole cohort of women and men, and the results were statistically significant in the fresh chilli group (fig 1 and appendix table 1).

**Figure fig1:**
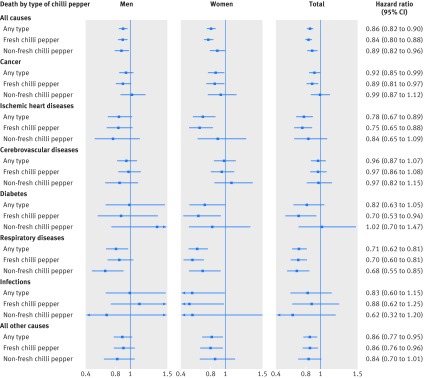
**Fig 1** Subgroup analysis of associations between consumption of spicy foods ≥6 days a week and total and cause specific mortality according to consumption of fresh chilli pepper. Hazard ratios for death from all causes and from specific causes are for comparison of men and women who ate spicy foods ≥6 days a week with those who ate spicy foods less than once a week. Appendix table 1 shows the risk estimates for other categories of spicy food consumption. Horizontal lines represent 95% confidence intervals

We also analyzed the associations between spicy food consumption and total mortality according to other potential baseline risk factors for death; the inverse associations between spicy food consumption and total mortality were generally similar across subgroups stratified according to age, smoking status, level of physical activity, and body mass index (all P values for interaction >0.05) (fig 2 and appendix table 2). Significant differences across stratums were observed for alcohol consumption, with a stronger inverse association among participants who did not consume alcohol than those who did (P=0.033 for interaction).

**Figure fig2:**
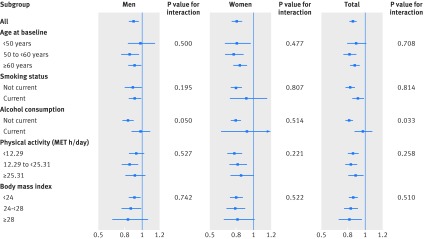
**Fig 2** Subgroup analysis of associations between consumption of spicy foods ≥6 days a week and total mortality according to potential baseline risk factors. Hazard ratios for total mortality are for comparison of men and women who ate spicy foods ≥6 days a week with those who ate spicy foods less than once a week. Risk estimates for other categories of spicy food consumption are shown in appendix table 2. Horizontal lines represent 95% confidence intervals

## Discussion

In this large prospective study, we observed an inverse association between consumption of spicy foods and total mortality, after adjusting for potential confounders. Compared with those who ate spicy foods less than once a week, those who consumed spicy foods almost every day had a 14% lower risk of death. Inverse associations were also observed for deaths due to cancer, ischemic heart diseases, and respiratory diseases. The associations were consistent in men and women.

### Strengths and limitations of this study

The strengths of this study include a large sample size, a prospective cohort design, and careful control for established and potential risk factors for death. This study does have a few limitations. Consumption of spicy foods may be correlated with other dietary habits and lifestyle behaviors. For example, in Chinese cuisine the cooking of chilli pepper and the production of chilli sauce and oil usually requires more oil, and intake of pungent foods may be accompanied by an increased intake of carbohydrate-rich foods such as rice to relieve the burning sensation. However, the lack of detailed dietary information in this study limited our ability to comprehensively adjust for total energy intake and other specific dietary factors. In addition, spicy food consumption may be correlated with socioeconomic status,^16^ which we partly controlled for in our analyses. Residual confounding by other unmeasured or unknown biological and social factors was still possible, although we carefully adjusted for several established and potential risk factors for death. However, residual confounding by the aforementioned or other confounding factors might have attenuated the inverse associations between spicy food consumption and mortality toward the null. Although chilli pepper was the most commonly used spice in our population, the use of other types of spices usually increases as the use of chilli pepper increases. Thus the health benefits of these spices apart from chilli pepper may also contribute to the observed inverse associations. Reverse causality is another possible explanation for our findings because people with chronic disease might abstain from spicy foods. However, we excluded participants who had cancer, heart disease, or stroke at baseline. Moreover, the results remained largely unchanged when we excluded participants dying during the first two years of follow-up from analyses or additionally adjusted for several major digestive system diseases that might deter people from consuming spicy foods.

Although we employed multiple ways to maximize death ascertainment of participants, under-reporting of deaths might have occurred. However, the proportion of participants under-reported on death status would not depend on the levels of spicy food consumption. Considering that specificity of outcome detection is nearly perfect and sensitivity is lower than 100% in both exposure groups, outcome misclassification would produce little bias in estimating hazard ratio.^17^ The consumption of spicy foods was self reported; therefore, some measurement error is inevitable. The questionnaire on spicy food consumption used in our study has not yet been validated directly; however, previous studies have shown that using similar food frequency questionnaires could produce valid estimates of food consumption in a Chinese population.^18^ In addition, in a prospective study design, measurement errors may be non-differential and the measure of association is more likely to be biased toward the null. The consumption of spicy foods reported for a short period may not necessarily reflect the long term patterns of consumption. However, repeated collections of dietary information averaged 1.4 years in our cohort and have shown that the reported intakes of dietary factors including spicy foods were highly consistent over time. In addition, information was not available on how spicy foods were prepared and cooked. Such information would have enabled us to perform further analyses on the relation between spicy food consumption and mortality more extensively.

### Comparison with other studies and potential mechanism

Our study is the first to analyze the association between daily consumption of spicy foods and mortality in a prospective cohort. Our findings are in line with previous evidence showing potential protective effects of spicy foods on human health. Capsaicin is the main active component of chilli pepper. The beneﬁcial roles of capsaicin have been extensively reported in relation to anti-obesity, antioxidant, anti-inflammatory, anticancer, and antihypertensive effects, and in improving glucose homeostasis, largely in experimental or small sized population studies.^7^ ^8^ ^9^ Additionally, the antimicrobial function of spices, including chilli pepper, has long been recognized,^3^ ^19^ and such a property may have an important effect on the gut microbiota in humans. In recent years, rapidly emerging evidence has implicated gut microbiota as a novel and important metabolic factor that affects the health of the host,^20^ and several studies in humans have related abundance, composition, and metabolites of gut microbiota to risk of obesity,^21^ ^22^ ^23^ diabetes,^12^ ^24^ liver cirrhosis,^11^ and cardiovascular disease.^10^ ^25^ However, how spicy foods and their bioactive ingredients may affect the composition and activity of gut microbiota has yet to be further investigated. In addition, our study suggested a threshold of around 1 or 2 days a week of spicy food consumption, beyond which the risk for mortality did not decrease further. Possible mechanisms might involve the bioaccessibility and bioavailability of bioactive ingredients and nutrients of spicy foods^26^; but further studies are needed to verify our findings. Our study indicated that spicy food consumption was particularly related to the reduced risk of mortality due to cancer, ischemic heart diseases, and respiratory diseases. Several previous epidemiological studies have suggested protective effects of capsaicin consumption on stomach or gallbladder cancer,^7^ ^9^ ^27^ although such effects were not consistently observed. The cardiovascular system is rich in capsaicin sensitive sensory nerves, which have an extensive role in regulating cardiovascular function.^28^ The antioxidant and antiplatelet properties of capsaicin and the important role of capsaicin in regulating energy metabolism may also contribute to its beneﬁcial effects on the cardiovascular system.^7^ ^9^ ^28^ ^29^ ^30^ ^31^ ^32^ Less well known are the possible mechanisms underlying the potentially beneficial effect of spicy foods on respiratory diseases. However, the anti-obesity, antioxidant, anti-inflammatory, and antihypertensive effects of spicy foods would generally protect all these specific systems. Because the number of deaths from infections was relatively small, our study might not have had enough statistical power to rule out a possible relation between spicy food consumption and infections specific mortality.

Compared with non-fresh spicy foods such as dried chilli pepper, chilli sauce, or chilli oil, fresh chilli pepper is richer in bioactive ingredients, including capsaicin, vitamin C, and other nutrients such as vitamin A, K, and B6, and potassium. In our stratified analyses we found that the inverse associations of spicy food consumption with certain cause specific deaths (cancer, ischemic heart disease, and diabetes) seemed to be stronger in those who consumed fresh chilli pepper than those who consumed non-fresh spicy foods. These data suggest that some of the bioactive ingredients are likely to be effective in driving the observed associations. Interestingly, a statistically significant inverse association between the daily consumption of spicy foods and diabetes, which was not observed in the whole cohort, was found in the subgroup that consumed fresh chilli pepper. This was consistent with previous evidence showing that dietary capsaicin may provide beneficial effects on glucose homeostasis.^33^ However, it remains unclear whether other nutrients abundant in fresh chilli pepper also have roles in lowering the risk of mortality. Intriguingly, we found that the inverse association was stronger in those who did not than did drink alcohol. Alcohol consumption has been related to an increased risk of mortality in some but not all previous studies.^34^ ^35^ ^36^ Even though moderate alcohol consumption has been related to a reduced risk of certain chronic diseases such as diabetes, moderately high alcohol consumption may increase energy intake^37^ and has been associated with increased mortality.^38^ In addition, alcohol intake also affects the metabolism of gut microbiota.^39^ ^40^ Even though the precise mechanism remains unclear, the interaction between spicy foods and alcohol intake is biologically possible. We acknowledge that disease status might affect both alcohol and spicy food intakes, and we excluded participants with chronic diseases such as cancer, heart disease, or stroke at baseline from our analyses. Further investigations are warranted to validate our findings and explore the mechanisms.

### Conclusion

Our analyses showed significant inverse associations between spicy food consumption and total and certain cause specific mortality (cancer, ischemic heart diseases, and respiratory diseases). None the less, given the observational nature of this study, it is not possible to make a causal inference. Further prospective studies in other populations would be essential to demonstrate generalizability of these findings. More evidence will lead to updated dietary recommendations and development of functional foods, such as herbal supplements.

**What is already known on this topic**

A beneficial role of spices and their major bioactive components has been reported in a variety of chronic disorders in experimental and small sized population studies
Evidence relating daily consumption of spicy foods to mortality from prospective cohort studies is lacking

**What this study adds**

The habitual consumption of spicy foods was inversely associated with total and certain cause specific mortality (cancer, ischemic heart diseases, and respiratory diseases), independent of other risk factors of death

## Notes

We thank the participants, project staff, and China National Center for Disease Control and Prevention (CDC) and its regional offices for access to death and disease registries. The Chinese National Health Insurance scheme provides electronic linkage to all hospital treatment.

China Kadoorie Biobank collaborative group: International steering committee: Liming Li (PI), Junshi Chen, Rory Collins, Richard Peto, Zhengming Chen (PI). Study coordinating centers: International Co-ordinating Center, Oxford: Zhengming Chen, Garry Lancaster, Xiaoming Yang, Alex Williams, Margaret Smith, Ling Yang, Yumei Chang, Iona Millwood, Yiping Chen, Sarah Lewington. National coordinating center, Beijing: Yu Guo, Jun Lv, Zheng Bian, Peng Liu, Canqing Yu, Pei Pei, Huiyan Zhou, Yunlong Tan, Can Hou, Lei Guo, Bingyang Han, Shuzhen Qu, Ge Chen. Regional coordinating centers, 10 areas in China: Qingdao CDC: Zengchang Pang, Shutao Pang, Shaojie Wang, Yongmei Liu, Ranran Du, Yajing Zang, Liang Cheng. Licang CDC: Silu Lv, Junzheng Wang, Wei Hou. Heilongjiang Provincial CDC: Jiyuan Yin, Shumei Liu, Zhigang Pang, Xue Zhou, Huijun Wang. Nangang CDC: Liqiu Yang, Bo Yu, Yanjie Li, Jing Qi, Huaiyi Mu, Qin’ai Xu, Meiling Dou. Hainan Provincial CDC: Jianwei Du, Shanqing Wang, Ximin Hu, Hongmei Wang, Jinyan Chen, Yan Fu, Zhenwang Fu, Xiaohuan Wang, Hua Dong. Meilan CDC: Min Weng, Xiangyang Zheng, Yijun Li, Huimei Li. Jiangsu Provincial CDC: Ming Wu, Jinyi Zhou, Ran Tao, Jie Yang. Suzhou CDC: Jie Shen, Yihe Hu, Yan Lu, Yan Gao, Liangcai Ma, Aiyu Tang, Shuo Zhang, Jianrong Jin. Guangxi Provincial CDC: Zhenzhu Tang, Naying Chen, Ying Huang. Liuzhou CDC: Mingqiang Li, Jinhuai Meng, Rong Pan, Qilian Jiang, Jingxin Qin, Weiyuan Zhang, Yun Liu, Liuping Wei, Liyuan Zhou, Ningyu Chen, Jun Yang, Hairong Guan. Sichuan Provincial CDC: Xianping Wu, Ningmei Zhang, Xiaofang Chen, Xuefeng Tang. Pengzhou CDC: Guojin Luo, Jianguo Li, Xiaofang Chen, Jian Wang, Jiaqiu Liu, Qiang Sun. Gansu Provincial CDC: Pengfei Ge, Xiaolan Ren, Caixia Dong. Maiji CDC: Hui Zhang, Enke Mao, Xiaoping Wang, Tao Wang. Henan Provincial CDC: Guohua Liu, Baoyu Zhu, Gang Zhou, Shixian Feng, Liang Chang, Lei Fan. Huixian CDC: Yulian Gao, Tianyou He, Li Jiang, Huarong Sun, Pan He, Chen Hu, Qiannan Lv, Xukui Zhang. Zhejiang Provincial CDC: Min Yu, Ruying Hu, Le Fang, Hao Wang. Tongxiang CDC: Yijian Qian, Chunmei Wang, Kaixu Xie, Lingli Chen, Yaxing Pan, Dongxia Pan. Hunan Provincial CDC: Yuelong Huang, Biyun Chen, Donghui Jin, Huilin Liu, Zhongxi Fu, Qiaohua Xu. Liuyang CDC: Xin Xu, Youping Xiong, Weifang Jia, Xianzhi Li, Libo Zhang, Zhe Qiu.

Contributors: JL and LQ are joint first authors. LL and ZC obtained funding. LL, ZC, and JC designed the study. YG, ZB, JD, PG, ZT, WH, YL, and ZC collected the data. LY and YC were involved in data cleaning, mortality follow-up, and verification. JL, CY, and DS analyzed the data. JL drafted the manuscript. LQ and LL contributed to the interpretation of the results and critical revision of the manuscript for important intellectual content and approved the final version of the manuscript. All authors have read and approved the final manuscript. LL and ZC are the study guarantors.

Ethical approval: This study was approved by the ethical review committee of the Chinese Center for Disease Control and Prevention (Beijing, China) and the Oxford Tropical Research Ethics Committee, University of Oxford (UK).

Data sharing: The access policy and procedures are available at www.ckbiobank.org.

Transparency: The lead authors (LL and ZC) affirm that the manuscript is an honest, accurate, and transparent account of the study being reported; that no important aspects of the study have been omitted; and that any discrepancies are disclosed.

## Notes

Cite this as: *BMJ* 2015;351:h3942
