# Supplementary material for: Consumption of spicy foods and total and cause specific mortality: population based cohort study
Source: BMJ. 2015 Aug 4;351:h3942. doi: 10.1136/bmj.h3942 (PMC4525189; doi:10.1136/bmj.h3942)
Supplement: Supplementary file 1 [file lvju025078.w1_default.pdf]

Supplementary Table 1. Association of weekly spicy food consumption with total and cause specific mortality according to the consumption of fresh chilli pepper

Cause of death	Type of chilli pepper	Less than once a week		1 or 2 days		3-5 days		6 or 7 days	
		No. of deaths	HR*	No. of deaths	HR* (95% CI)	No. of deaths	HR* (95% CI)	No. of deaths	HR* (95% CI)
**Total**									
**No. of person years**	Any type	1,990,589		228,112		204,972		1,076,330	
	Fresh			139,422		140,097		949,250	
	Non-fresh			88,691		64,875		127,081	
**All causes**	Any type	12,145	1.00	1,014	0.90 (0.84-0.96)	876	0.86 (0.80-0.92)	6,189	0.86 (0.82-0.90)
	Fresh		1.00	626	0.92 (0.84-1.00)	572	0.82 (0.75-0.89)	5,305	0.84 (0.80-0.88)
	Non-fresh		1.00	388	0.85 (0.77-0.95)	304	0.92 (0.82-1.03)	884	0.89 (0.82-0.96)
**Cancer**	Any type	4,636	1.00	378	0.92 (0.83-1.03)	349	0.97 (0.87-1.08)	1,893	0.92 (0.85-0.99)
	Fresh		1.00	223	0.93 (0.81-1.07)	210	0.88 (0.77-1.02)	1,565	0.89 (0.81-0.97)
	Non-fresh		1.00	155	0.89 (0.76-1.05)	139	1.11 (0.94-1.32)	328	0.99 (0.87-1.12)
**Ischemic heart diseases**	Any type	1,406	1.00	109	0.82 (0.67-1.00)	93	0.75 (0.61-0.94)	694	0.78 (0.67-0.89)
	Fresh		1.00	78	0.81 (0.64-1.02)	64	0.67 (0.52-0.87)	623	0.75 (0.65-0.88)
	Non-fresh		1.00	31	0.82 (0.57-1.17)	29	0.99 (0.68-1.44)	71	0.84 (0.65-1.09)
**Cerebrovascular diseases**	Any type	2,217	1.00	215	1.03 (0.89-1.20)	170	0.86 (0.73-1.01)	1,422	0.96 (0.87-1.07)
	Fresh		1.00	140	1.08 (0.90-1.29)	119	0.85 (0.70-1.03)	1250	0.97 (0.86-1.08)
	Non-fresh		1.00	75	0.97 (0.77-1.23)	51	0.89 (0.67-1.18)	172	0.97 (0.82-1.15)
**Diabetes**	Any type	328	1.00	30	0.94 (0.64-1.40)	17	0.60 (0.36-0.99)	194	0.82 (0.63-1.05)
	Fresh		1.00	13	0.66 (0.37-1.18)	12	0.62 (0.34-1.13)	154	0.70 (0.53-0.94)
	Non-fresh		1.00	17	1.34 (0.81-2.21)	5	0.50 (0.21-1.23)	40	1.02 (0.70-1.47)

Cause of death	Type of chilli pepper	Less than once a week		1 or 2 days		3-5 days		6 or 7 days	
		No. of deaths	HR*	No. of deaths	HR* (95% CI)	No. of deaths	HR* (95% CI)	No. of deaths	HR* (95% CI)
**Respiratory diseases**	Any type	1,203	1.00	77	0.67 (0.53-0.85)	70	0.65 (0.51-0.83)	646	0.71 (0.62-0.81)
	Fresh		1.00	45	0.75 (0.55-1.02)	46	0.69 (0.51-0.93)	540	0.70 (0.60-0.81)
	Non-fresh		1.00	32	0.57 (0.40-0.81)	24	0.56 (0.37-0.85)	106	0.68 (0.55-0.85)
**Infections**	Any type	187	1.00	20	0.91 (0.56-1.48)	15	0.74 (0.43-1.28)	114	0.83 (0.60-1.15)
	Fresh		1.00	15	1.07 (0.61-1.87)	12	0.83 (0.45-1.53)	103	0.88 (0.62-1.25)
	Non-fresh		1.00	5	0.64 (0.26-1.58)	3	0.52 (0.16-1.63)	11	0.62 (0.32-1.20)
**All other causes**	Any type	2,168	1.00	185	0.89 (0.77-1.04)	162	0.89 (0.75-1.05)	1,226	0.86 (0.77-0.95)
	Fresh		1.00	112	0.94 (0.77-1.15)	109	0.92 (0.75-1.12)	1070	0.86 (0.76-0.96)
	Non-fresh		1.00	73	0.83 (0.66-1.06)	53	0.85 (0.64-1.11)	156	0.84 (0.70-1.01)
**Men**									
**No. of person years**	Any type	783,656		101,892		90,478		440,002	
	Fresh			63,259		62,330		390,538	
	Non-fresh			38,633		28,148		49,464	
**All causes**	Any type	6,872	1.00	606	0.90 (0.83-0.98)	545	0.90 (0.83-0.99)	3797	0.90 (0.85-0.96)
	Fresh		1.00	386	0.94 (0.85-1.05)	355	0.85 (0.76-0.95)	3333	0.90 (0.84-0.96)
	Non-fresh		1.00	220	0.83 (0.73-0.96)	190	1.00 (0.86-1.16)	464	0.88 (0.79-0.98)
**Cancer**	Any type	2,769	1.00	230	0.88 (0.77-1.01)	236	1.05 (0.92-1.21)	1188	0.94 (0.85-1.04)
	Fresh		1.00	141	0.94 (0.79-1.12)	140	0.95 (0.80-1.14)	988	0.90 (0.81-1.01)
	Non-fresh		1.00	89	0.81 (0.65-1.00)	96	1.24 (1.00-1.52)	200	1.02 (0.87-1.20)
**Ischemic heart**	Any type	745	1.00	66	0.87 (0.67-1.13)	60	0.85 (0.65-1.11)	417	0.85 (0.70-1.02)
	Fresh		1.00	46	0.81 (0.60-1.10)	43	0.76 (0.56-1.05)	384	0.84 (0.69-1.03)

Cause of death	Type of chilli pepper	Less than once a week		1 or 2 days		3-5 days		6 or 7 days	
		No. of deaths	HR*	No. of deaths	HR* (95% CI)	No. of deaths	HR* (95% CI)	No. of deaths	HR* (95% CI)
**diseases**	Non-fresh		1.00	20	1.01 (0.64-1.58)	17	1.09 (0.67-1.78)	33	0.77 (0.52-1.13)
**Cerebrovascular diseases**	Any type	1,199	1.00	117	1.00 (0.82-1.22)	85	0.77 (0.62-0.97)	821	0.95 (0.83-1.09)
	Fresh		1.00	77	1.03 (0.81-1.31)	62	0.78 (0.60-1.02)	745	0.98 (0.84-1.14)
	Non-fresh		1.00	40	0.97 (0.70-1.33)	23	0.74 (0.48-1.13)	76	0.86 (0.66-1.10)
**Diabetes**	Any type	116	1.00	13	1.10 (0.60-2.01)	7	0.71 (0.33-1.55)	95	0.99 (0.67-1.46)
	Fresh		1.00	7	0.85 (0.38-1.92)	7	1.00 (0.45-2.20)	79	0.88 (0.56-1.37)
	Non-fresh		1.00	6	1.51 (0.65-3.52)	0	--	16	1.36 (0.75-2.47)
**Respiratory diseases**	Any type	706	1.00	51	0.79 (0.59-1.06)	38	0.61 (0.44-0.86)	422	0.81 (0.67-0.97)
	Fresh		1.00	29	0.80 (0.55-1.18)	25	0.62 (0.41-0.93)	373	0.85 (0.70-1.04)
	Non-fresh		1.00	22	0.75 (0.49-1.16)	13	0.57 (0.33-0.99)	49	0.66 (0.48-0.91)
**Infections**	Any type	116	1.00	13	0.91 (0.50-1.65)	11	0.83 (0.44-1.58)	82	0.99 (0.67-1.48)
	Fresh		1.00	9	0.98 (0.48-2.00)	9	0.96 (0.47-1.95)	76	1.12 (0.74-1.71)
	Non-fresh		1.00	4	0.85 (0.31-2.33)	2	0.55 (0.14-2.27)	6	0.68 (0.29-1.63)
**All other causes**	Any type	1,221	1.00	116	0.92 (0.75-1.11)	108	0.97 (0.79-1.19)	772	0.89 (0.78-1.03)
	Fresh		1.00	77	1.04 (0.82-1.32)	69	0.93 (0.72-1.20)	688	0.91 (0.78-1.06)
	Non-fresh		1.00	39	0.75 (0.54-1.03)	39	1.05 (0.76-1.45)	84	0.83 (0.65-1.06)
**Women**									
**No. of person years**	Any type	1,206,933		126,220		114,494		636,329	
	Fresh			76,162		77,767		558,712	
	Non-fresh			50,058		36,727		77,617	
**All causes**	Any type	5,273	1.00	408	0.88 (0.79-0.98)	331	0.78 (0.69-0.88)	2392	0.81 (0.75-0.87)

Cause of death	Type of chilli pepper	Less than once a week		1 or 2 days		3-5 days		6 or 7 days	
		No. of deaths	HR*	No. of deaths	HR* (95% CI)	No. of deaths	HR* (95% CI)	No. of deaths	HR* (95% CI)
	Fresh		1.00	240	0.88 (0.77-1.00)	217	0.77 (0.67-0.89)	1972	0.77 (0.71-0.83)
	Non-fresh		1.00	168	0.87 (0.74-1.01)	114	0.78 (0.65-0.94)	420	0.89 (0.80-0.99)
**Cancer**	Any type	1,867	1.00	148	0.97 (0.82-1.15)	113	0.82 (0.68-1.00)	705	0.87 (0.77-0.99)
	Fresh		1.00	82	0.91 (0.72-1.14)	70	0.77 (0.60-0.98)	577	0.86 (0.75-0.98)
	Non-fresh		1.00	66	1.03 (0.80-1.33)	43	0.90 (0.66-1.22)	128	0.94 (0.77-1.15)
**Ischemic heart diseases**	Any type	661	1.00	43	0.74 (0.54-1.02)	33	0.63 (0.44-0.90)	277	0.70 (0.56-0.86)
	Fresh		1.00	32	0.79 (0.55-1.15)	21	0.53 (0.34-0.83)	239	0.65 (0.51-0.83)
	Non-fresh		1.00	11	0.60 (0.33-1.10)	12	0.86 (0.48-1.54)	38	0.90 (0.63-1.28)
**Cerebrovascular diseases**	Any type	1,018	1.00	98	1.08 (0.87-1.34)	85	0.96 (0.76-1.21)	601	0.98 (0.85-1.14)
	Fresh		1.00	63	1.16 (0.89-1.52)	57	0.93 (0.70-1.23)	505	0.95 (0.81-1.12)
	Non-fresh		1.00	35	0.96 (0.68-1.36)	28	1.02 (0.69-1.49)	96	1.08 (0.86-1.37)
**Diabetes**	Any type	212	1.00	17	0.87 (0.52-1.46)	10	0.54 (0.28-1.04)	99	0.72 (0.52-1.01)
	Fresh		1.00	6	0.55 (0.23-1.28)	5	0.43 (0.17-1.06)	75	0.64 (0.43-0.94)
	Non-fresh		1.00	11	1.26 (0.67-2.36)	5	0.66 (0.27-1.64)	24	0.83 (0.51-1.33)
**Respiratory diseases**	Any type	497	1.00	26	0.51 (0.34-0.76)	32	0.69 (0.48-1.00)	224	0.62 (0.51-0.76)
	Fresh		1.00	16	0.69 (0.42-1.15)	21	0.83 (0.53-1.30)	167	0.56 (0.44-0.71)
	Non-fresh		1.00	10	0.35 (0.18-0.65)	11	0.53 (0.29-0.96)	57	0.69 (0.51-0.94)
**Infections**	Any type	71	1.00	7	0.86 (0.38-1.96)	4	0.55 (0.19-1.56)	32	0.55 (0.31-0.99)
	Fresh		1.00	6	1.20 (0.48-2.98)	3	0.60 (0.18-1.99)	27	0.52 (0.27-0.98)
	Non-fresh		1.00	1	0.31 (0.04-2.30)	1	0.41 (0.06-3.04)	5	0.56 (0.21-1.50)
**All other**	Any type	947	1.00	69	0.85 (0.66-1.10)	54	0.76 (0.58-1.02)	454	0.82 (0.69-0.96)

Cause of death	Type of chilli pepper	Less than once a week		1 or 2 days		3-5 days		6 or 7 days	
		No. of deaths	HR*	No. of deaths	HR* (95% CI)	No. of deaths	HR* (95% CI)	No. of deaths	HR* (95% CI)
**causes**	Fresh		1.00	35	0.77 (0.54-1.10)	40	0.90 (0.65-1.25)	382	0.80 (0.67-0.96)
	Non-fresh		1.00	34	0.95 (0.67-1.35)	14	0.54 (0.32-0.92)	72	0.86 (0.66-1.13)

*Risk estimates: hazard ratio (95% confidence interval). Multivariate models were adjusted for the following baseline factors: age (years); sex (male or female, only for the whole cohort); level of education (no formal school, primary school, middle school, high school, college, or university or higher); marital status (married, widowed, divorced or separated, or never married); alcohol consumption (non-drinker, occasional drinker, former drinker, or regular drinker); smoking status (never smoker, occasional smoker, former smoker, or regular smoker); physical activity (MET (metabolic equivalent of task) h/day); body mass index; intake frequencies of red meat, fruits, and vegetables (daily, 4-6 days/week, 1-3 days/week, monthly, or rarely/never); prevalent hypertension and diabetes (presence or absence); family history of cancer, heart attack, stroke, or diabetes (presence or absence, only adjusted for in the corresponding analysis of cause-specific mortality); and menopausal status (premenopausal, perimenopausal, or postmenopausal, for women only).

Supplementary Table 2. Association of weekly spicy food consumption with total mortality by baseline subgroup*

Subgroups	Less than once a week			1 or 2 days			3-5 days			6 or 7 days			P value for interaction***
	No. of person years	No. of deaths	HR**	No. of person years	No. of deaths	HR** (95% CI)	No. of person years	No. of deaths	HR** (95% CI)	No. of person years	No. of deaths	HR** (95% CI)	
**Total**													
All	1,990,589	12,145	1.00	228,112	1,014	0.90 (0.84-0.96)	204,972	876	0.86 (0.80-0.92)	1,076,330	6,189	0.86 (0.82-0.90)	
Age < 50 yr	856,287	1,323	1.00	130,772	185	0.89 (0.76-1.05)	118,438	169	0.87 (0.73-1.02)	560,550	1,054	0.90 (0.80-1.01)	
50 to < 60 yr	634,369	2,794	1.00	62,957	265	0.84 (0.74-0.96)	56,741	254	0.88 (0.77-1.00)	323,265	1,727	0.82 (0.75-0.90)	0.708
≥ 60 yr	499,933	8,028	1.00	34,383	564	0.94 (0.86-1.02)	29,794	453	0.85 (0.77-0.93)	192,516	3,408	0.88 (0.83-0.93)	
Not regular smoker	1,526,472	8,065	1.00	158,893	571	0.86 (0.79-0.94)	141,116	479	0.82 (0.75-0.91)	744,385	3,242	0.83 (0.78-0.88)	0.814
Regular smoker	464,117	4,080	1.00	69,220	443	0.95 (0.86-1.05)	63,856	397	0.91 (0.82-1.01)	331,946	2,947	0.92 (0.85-0.98)	
Not regular drinker	1,732,972	10,347	1.00	182,689	813	0.88 (0.81-0.95)	161,413	671	0.80 (0.74-0.87)	893,804	4,748	0.82 (0.78-0.86)	0.033
Regular drinker	257,617	1,798	1.00	45,424	201	0.91 (0.79-1.06)	43,559	205	1.00 (0.86-1.15)	182,526	1,441	0.97 (0.88-1.08)	
MET 0-	686,836	6,721	1.00	51,361	389	0.86 (0.78-0.96)	45,366	360	0.90 (0.81-1.00)	303,269	2,890	0.86 (0.80-0.92)	
MET 12.29-	636,634	3,095	1.00	78,707	333	0.93 (0.83-1.04)	72,346	264	0.78 (0.69-0.89)	402,423	2,001	0.84 (0.77-0.91)	0.258
MET 25.31-	667,119	2,329	1.00	98,044	292	0.94 (0.83-1.07)	87,260	252	0.91 (0.79-1.04)	370,638	1,298	0.89 (0.81-0.98)	
BMI <24.0	1,082,545	7,632	1.00	123,430	633	0.87 (0.80-0.95)	109,335	539	0.82 (0.75-0.89)	674,828	4,477	0.86 (0.82-0.91)	
BMI 24.0 to < 28.0	685,210	3,348	1.00	78,757	275	0.91 (0.80-1.03)	71,163	254	0.92 (0.81-1.05)	316,720	1,345	0.83 (0.76-0.91)	0.510
BMI ≥ 28.0	222,834	1,165	1.00	25,925	106	0.99 (0.81-1.22)	24,475	83	0.84 (0.67-1.06)	84,764	366	0.82 (0.70-0.96)	
**Men**													
All	783,656	6,872	1.00	101,892	606	0.90 (0.83-0.98)	90,478	545	0.90 (0.83-0.99)	440,002	3,797	0.90 (0.85-0.96)	

Subgroups	Less than once a week			1 or 2 days			3-5 days			6 or 7 days			P value for interaction***
	No. of person years	No. of deaths	HR**	No. of person years	No. of deaths	HR** (95% CI)	No. of person years	No. of deaths	HR** (95% CI)	No. of person years	No. of deaths	HR** (95% CI)	
Age < 50 yr	326,692	741	1.00	56,318	112	0.90 (0.73-1.10)	50,498	104	0.89 (0.72-1.11)	206,102	613	0.98 (0.84-1.15)	
50 to < 60 yr	241,896	1,559	1.00	28,366	148	0.79 (0.67-0.94)	25,379	147	0.88 (0.74-1.04)	137,105	1,040	0.86 (0.76-0.96)	0.500
≥ 60 yr	215,069	4,572	1.00	17,207	346	0.97 (0.86-1.08)	14,601	294	0.93 (0.82-1.05)	96,795	2,144	0.91 (0.85-0.99)	
Not regular smoker	338,382	3,075	1.00	35,424	188	0.80 (0.69-0.93)	29,788	177	0.90 (0.77-1.05)	130,128	1,069	0.90 (0.81-0.99)	0.195
Regular smoker	445,274	3,797	1.00	66,468	418	0.96 (0.87-1.07)	60,690	368	0.92 (0.82-1.03)	309,874	2,728	0.92 (0.85-0.99)	
Not regular drinker	541,734	5,147	1.00	59,732	418	0.89 (0.80-0.98)	50,580	349	0.84 (0.75-0.94)	278,666	2,467	0.84 (0.78-0.90)	0.050
Regular drinker	241,922	1,725	1.00	42,160	188	0.89 (0.77-1.04)	39,898	196	1.00 (0.86-1.16)	161,336	1,330	0.98 (0.88-1.09)	
MET 0-	249,223	3,666	1.00	20,934	228	0.92 (0.80-1.05)	18,305	216	0.98 (0.85-1.12)	125,737	1,756	0.93 (0.85-1.02)	
MET 12.29-	219,190	1,648	1.00	35,279	195	0.87 (0.75-1.02)	31,814	174	0.84 (0.72-0.99)	150,167	1,221	0.86 (0.77-0.95)	0.527
MET 25.31-	315,243	1,558	1.00	45,679	183	0.94 (0.80-1.10)	40,359	155	0.90 (0.76-1.07)	164,098	820	0.92 (0.81-1.04)	
BMI < 24.0	439,098	4,632	1.00	53,896	405	0.90 (0.81-1.00)	47,506	344	0.85 (0.76-0.95)	291,628	2,911	0.91 (0.85-0.98)	
BMI 24.0 to < 28.0	271,686	1,753	1.00	36,275	154	0.91 (0.77-1.07)	32,262	157	1.04 (0.88-1.23)	120,219	719	0.87 (0.77-0.99)	0.742
BMI ≥ 28.0	72,873	487	1.00	11,721	47	0.84 (0.62-1.15)	10,711	44	0.85 (0.62-1.17)	28,146	167	0.84 (0.66-1.07)	
**Women**													
All	1,206,933	5,273	1.00	126,220	408	0.88 (0.79-0.98)	114,494	331	0.78 (0.69-0.88)	636,329	2,392	0.81 (0.75-0.87)	
Age < 50 yr	529,595	582	1.00	74,454	73	0.89 (0.69-1.16)	67,939	65	0.83 (0.64-1.09)	354,448	441	0.81 (0.67-0.97)	
50 to < 60 yr	392,473	1,235	1.00	34,591	117	0.88 (0.72-1.07)	31,362	107	0.86 (0.70-1.06)	186,159	687	0.77 (0.68-0.88)	0.477
≥ 60 yr	284,865	3,456	1.00	17,176	218	0.89 (0.77-1.02)	15,193	159	0.72 (0.61-0.85)	95,721	1,264	0.84 (0.77-0.92)	

Subgroups	Less than once a week			1 or 2 days			3-5 days			6 or 7 days			P value for interaction[***]
	No. of person years	No. of deaths	HR[**]	No. of person years	No. of deaths	HR[**] (95% CI)	No. of person years	No. of deaths	HR[**] (95% CI)	No. of person years	No. of deaths	HR[**] (95% CI)	
Not regular smoker	1,188,090	4,990	1.00	123,469	383	0.89 (0.79-0.99)	111,327	302	0.78 (0.69-0.88)	614,257	2,173	0.80 (0.74-0.86)	0.807
Regular smoker	18,843	283	1.00	2,752	25	0.81 (0.53-1.23)	3,167	29	0.80 (0.53-1.19)	22,072	219	0.92 (0.73-1.15)	
Not regular drinker	1,191,238	5,200	1.00	122,956	395	0.87 (0.78-0.96)	110,834	322	0.77 (0.69-0.87)	615,138	2,281	0.80 (0.74-0.85)	0.514
Regular drinker	15,695	73	1.00	3,264	13	1.27 (0.69-2.36)	3,660	9	0.97 (0.47-1.99)	21,191	111	0.92 (0.63-1.35)	
MET 0-	437,613	3,055	1.00	30,427	161	0.78 (0.66-0.92)	27,061	144	0.80 (0.67-0.94)	177,532	1,134	0.78 (0.70-0.87)	
MET 12.29-	417,445	1,447	1.00	43,428	138	1.03 (0.86-1.24)	40,532	90	0.68 (0.55-0.85)	252,257	780	0.82 (0.72-0.92)	0.221
MET 25.31-	351,876	771	1.00	52,365	109	0.93 (0.75-1.15)	46,901	97	0.90 (0.72-1.13)	206,540	478	0.86 (0.74-1.00)	
BMI 18.5 to < 24.0	643,448	3,000	1.00	69,534	228	0.81 (0.71-0.93)	61,829	195	0.75 (0.65-0.88)	383,201	1,566	0.80 (0.73-0.88)	
BMI 24.0 to < 28.0	413,524	1,595	1.00	42,483	121	0.90 (0.75-1.09)	38,901	97	0.78 (0.63-0.97)	196,502	626	0.79 (0.69-0.90)	0.522
BMI ≥ 28.0	149,961	678	1.00	14,204	59	1.15 (0.87-1.52)	13,764	39	0.82 (0.59-1.15)	56,618	199	0.82 (0.66-1.01)	

[*] Body mass index (BMI) was missing for two participants.

[**] Risk estimates: hazard ratio (95% confidence interval). Multivariate models were adjusted for the following baseline factors: age (years); sex (male or female, only for the whole cohort); level of education (no formal school, primary school, middle school, high school, college, or university or higher); marital status (married, widowed, divorced or separated, or never married); alcohol consumption (non-drinker, occasional drinker, former drinker, or regular drinker); smoking status (never smoker, occasional smoker, former smoker, or regular smoker); physical activity (MET (metabolic equivalent of task) h/day); body mass index; intake frequencies of red meat, fruits, and vegetables (daily, 4-6 days/week, 1-3 days/week, monthly, or rarely/never); prevalent hypertension and diabetes (presence or absence); and menopausal status (premenopausal, perimenopausal, or postmenopausal, for women only). The level of physical activity was categorized by tertile cut-offs.

[***] The P values for interaction were computed with the use of likelihood-ratio tests comparing models with and without cross-product terms between the baseline stratifying variable and spicy food consumption as an ordinal variable.
